# Supplementary material for: Perceived benefits and challenges one year after receiving brief therapy in a district psychiatric centre. An exploration of patients’ and GPs’ experiences: A qualitative study
Source: PLoS One. 2024 Dec 23;19(12):e0312513. doi: 10.1371/journal.pone.0312513 (PMC11666029; doi:10.1371/journal.pone.0312513)
Supplement: Attachment — Submitted filename: Review Manuscript Number PONE-D-24-03765.docx [file pone.0312513.s002.docx]

***Review:***

**Manuscript Number PONE-D-24-03765
"Who benefits from brief therapy for mental health problems in the long term? An exploration of patients' and general practitioners' experiences: A Qualitative Study"**

Thank you for letting me review this interesting manuscript of a very important subject. I think the work is ambitious and to most parts well described with reasonable conclusions. I would recommend the authors to attach a Checklist of qualitative studies such as the COREQ, because there are some questions there which could be more clearly described, e.g. the relationships of the researchers to the participating, patients, GPs and to the project?

**Introduction:**

It was a bit hard to find out what the definition of “brief therapy” is. In Primary Care it could mean 3-4 sessions, but here it is unclear, in p13:90 4-26 sessions are mentioned? On p 12:82-84 there is also no difference mentioned between what is considered brief PDT compared to CBT, in the latter 12 sessions are considered standard, not brief.

Later in the manuscript the included patients received 8-15 sessions of either CBT or MCT, which is standard for these treatments not brief. Kind of therapy and if diagnose-specific or unified protocols were used? Needs clarification.

**Methods:**

The diagnostic procedure seems lacking, including severity assessment, which the authors also conclude could be a major cause of the findings. Also makes me wonder about the design of the project, e.g. treatment for PTSD is specific CBT, prolonged exposure (PE) according to guidelines and research evidence, why give these patients some brief intervention based on any diagnosis of anxiety or depression? One size fits all?

I guess it relates to the previous study but to be able to interpret the qualitative interviews it is important to know: When and by whom were the assessments made, which kind of treatment protocols were used? Inclusion, exclusion criteria? Pharmaceutical treatment? Protocol if patient got worse, suicidal? Because there seems to be a mix of conditions, these factors are important to interpret the results.

**Qualitative methods:**

P14:133: Recruitment and selection of participants: Out of 459 treated patients 11 participated in the interviews. How many were invited? How did the authors secure a trustworthy generalizable sample? Was there a defined saturation point of variability from the interviews?

P14:139; P15:145: Selected by purposeful criterion sampling, please clarify, what criteria?

Procedure, analysis: The interviews and processes are well described but the thematic analysis and coding process remains unclear. Are the three different “stories” categories or themes? Subcategories?

P18:220 description of meaning units and coding in groups more similar to content analysis? To me the analysis process remains unclear.

**Results:**

Table 2: Almost seems like a quantitative analysis and report of how many patients were in the three groups (“stories”). In that case we lack the power of a quantative analysis with just 11 participants with very different kind of conditions and experiences of previous care according to table 1. Makes it hard to ensure transferability and generalizability.

Good and clarifying to see quotations of each story, but to ensure trustworthiness of the coding process a table or some example of coding the meaning units would clarify.

**Limitations:**

As described, pre-understanding can be positive but could also affect the interpretation and analysis if not made aware. Please describe researchers relationship to the material, and clarify how the close-to-text-analysis was performed.

As mentioned above there are several limitations to the recruitment and analysis which means difficulties to generalize the findings to all patients and GPs in the project, but also to other health care in general.

**Conclusions:**

The conclusions seem adequate and obvious in this context, but if “brief intervention” can stand for a mix of interventions (CBT, MCT) of 8-15 sessions and for different conditions, with different complexity and severity it is hard to draw any further conclusion of the intervention. The participants in story A maybe could have been helped with an even briefer intervention or maybe a group or an internet mediated intervention, whereas we know that people with bipolar, PTSD or other severe conditions need specific interventions.

But this is important knowledge if not known before. P32:572 assessment of main diagnosis, comorbidity and severity is necessary to refer patients to correct level and kind of care.
